# Chronic military stress and glandular epithelial tumor biology: an integrative neuroendocrine–inflammatory framework with insights from microgravity gene discovery

**DOI:** 10.3389/fonc.2026.1817973

**Published:** 2026-06-09

**Authors:** David Laván, Natalia Argüelles, Rosa Rea, José Morales, Sofia Montes, Daniel Huaman, Alexis Lluncor, Juan Moyano, Milton Peña, Vilma Herencia-Reyes, Alcides Guerra, Gabriela Calderón, José M. Vela-Ruiz, Aly Gallo

**Affiliations:** 1 Instituto de Investigaciones en Ciencias Biomédicas, Facultad de Medicina Humana, Universidad Ricardo Palma, Lima, Peru; 2 Department of Research, Development and Innovation, BioTechCell Sociedad Anónima Cerrada (SAC), Lima, Peru; 3 Faculty of Natural and Mathematical Sciences, National University Federico Villareal, Lima, Peru; 4 Escuela de Biología, Facultad de Ciencias Biológicas, Universidad Ricardo Palma, Lima, Peru; 5 Escuela de Medicina Humana, Universidad Señor de Sipán, Chiclayo, Peru; 6 Escuela de Ingeniería Electrónica, Facultad de Ingeniería, Universidad Ricardo Palma, Lima, Peru; 7 Escuela de Medicina Humana, Facultad de Medicina Humana, Universidad Ricardo Palma, Lima, Peru; 8 Department of International Clinical Oncology, Clínica Internacional, Lima, Peru; 9 Center for Research in Precision Medicine, Genomics, and Human Performance, Lima, Peru

**Keywords:** BDNF, chronic stress, epithelial-mesenchymal transition, FKBP5, glandular epithelial neoplasms, hypothalamic-pituitary-adrenal axis, IL-6, military personnel

## Abstract

Exposure to extreme stress within military contexts such as combat, captivity, survival training, or blast exposure triggers complex neurobiological responses that, in susceptible individuals, culminate in conditions such as Post-Traumatic Stress Disorder (PTSD). This inter-individual variability is rooted in profound genetic and epigenetic foundations. This manuscript reviews the critical relationship between chronic military stress and five key molecules: the glucocorticoid receptor (NR3C1), the FK506-binding protein 5 (FKBP5), brain-derived neurotrophic factor (BDNF), neuropeptide Y (NPY), and interleukin-6 (IL6). We examine how the dysregulation of this allostatic network predisposes individuals to PTSD and generates an altered systemic inflammatory and neuroendocrine microenvironment. Seeking an integrative biological perspective, this pathogenic model is linked to discoveries derived from extreme physical environments. Previous investigations by our group involving *Drosophila melanogaster* exposed to microgravity identified genes that are differentially inhibited under spaceflight-induced stress. Interactomic and evolutionary homology analyses revealed that five of these genes (LDHA, DNAJB5, ELOVL1, CLEC4M, SLC17A5) represent oncogenic vulnerabilities in human glandular epithelial tumors. Notably, network analysis demonstrates that *LDHA* and *DNAJB5* act as primary convergence nodes that interact directly with the allostatic stress network. We propose that the systemic attrition provoked by military stress acts as the physiological trigger that exploits these evolutionarily conserved epithelial vulnerabilities, thereby facilitating neoplastic progression. Understanding this translational convergence is fundamental for the development of predictive biomarkers and targeted therapies in high-risk populations.

## 1 Introduction

Military service exposes individuals to levels of psychological and physical stress that rarely find a parallel in civilian settings. Operational demands, combat exposure, prolonged separation from support networks, and hostile environments impose sustained challenges on the body’s allostatic regulatory systems (1). While many service members develop adaptive responses, a significant proportion experience persistent biological and psychological alterations, with Post-Traumatic Stress Disorder (PTSD) representing the most prevalent and studied outcome (2). Increasing evidence supports the conceptualization of PTSD not as an exclusively psychiatric condition, but as a systemic syndrome involving coordinated dysregulation of neuroendocrine, immune, autonomic, and potentially oncogenic pathways (3, 4).

In parallel, oncological epidemiology in military and veteran populations reveals consistent patterns that extend beyond conventional risk factors. Prostate cancer remains the most prevalent solid malignancy among male veterans, with a substantial clinical burden documented by the Veterans Health Administration (5). Lung and bronchial cancers, associated with high rates of smoking and occupational exposures such as hydrocarbon combustion and particulate matter, continue to show elevated incidence, prompting the implementation of low dose computed tomography screening programs (6, 7). Colorectal cancer presents disparities in diagnosis and access to care within the VA system (8–10), while cutaneous melanoma is linked to cumulative solar exposure during training and deployment (11–13). In younger populations, testicular tumors and their long-term sequelae have been described (14). Among female service members and veterans, breast cancer is the most common malignancy, with documented disparities in outcomes and access to care (15–17), while cervical and thyroid cancers, including those associated with exposures such as Agent Orange, complete the prevalent oncological spectrum (18–20).

The coexistence of chronic stress burden and increased incidence of glandular epithelial neoplasms particularly hepatocellular carcinoma (HCC), pancreatic ductal adenocarcinoma (PDAC), and prostatic adenocarcinoma suggests the presence of shared biological mechanisms. These tumors are highly sensitive to endocrine, inflammatory, and metabolic signals, all of which are profoundly altered under chronic stress conditions. This convergence supports the hypothesis that sustained activation of the hypothalamic–pituitary–adrenal (HPA) axis and its downstream molecular mediators may constitute a previously underrecognized pathogenic axis in oncogenesis.

At the molecular level, several key regulators emerge at the interface between chronic stress and tumor biology. The NR3C1 gene, encoding the glucocorticoid receptor (GR), functions as a ligand-dependent transcription factor controlling metabolic, immune, and proliferative gene networks (21). Chronic glucocorticoid exposure induces persistent transcriptional activation and epigenetic remodeling, particularly in hormonally regulated tissues such as liver and prostate (22). The *FKBP5* gene encodes a co-chaperone that modulates glucocorticoid receptor (GR) sensitivity through a rapid feedback loop which, under conditions of chronic stress and genetic susceptibility, can lead to sustained overexpression (23, 24). Beyond glucocorticoid regulation, FKBP5 also influences the PI3K/AKT signaling pathway, thereby affecting key cellular processes such as proliferation, apoptosis, and therapeutic resistance (25, 26). In this context, dysfunction of glucocorticoid signaling not only disrupts endocrine homeostasis but also promotes an environment conducive to the activation of inflammatory pathways. Among these, the IL-6/STAT3 axis emerges as a central mechanism linking systemic inflammation to tumor-promoting transcriptional programs. Interleukin-6, chronically elevated in stress and post-traumatic stress disorder (PTSD) (27, 28), activates JAK/STAT3 signaling, thereby promoting proliferation, epithelial–mesenchymal transition (EMT), and metabolic reprogramming (29–31). This pathway can generate autocrine inflammatory loops associated with metastatic potential and cancer stem cell phenotypes (32). In parallel, brain-derived neurotrophic factor (BDNF), traditionally associated with neuronal plasticity, has been implicated in epithelial tumor progression through activation of the RAS/MAPK pathway via TrkB, enhancing survival and therapeutic resistance (33–35). Its expression is significantly altered under chronic stress, including epigenetic modifications such as promoter hypermethylation (36). Neuropeptide Y (NPY), a key modulator of the sympathetic response, regulates vascular tone and angiogenesis through Y1 and Y2 receptors (37, 38) and may contribute to tumor microenvironment remodeling under sustained stress conditions. Importantly, this pathway is pharmacologically targetable, suggesting potential therapeutic implications (39).

In this context, the involvement of NPY is integrated within a broader network of neuroendocrine and inflammatory regulation. Collectively, these mechanisms support an integrative model in which chronic activation of the HPA axis induces sustained hypercortisolemia, persistent NR3C1 signaling, and FKBP5 overexpression, leading to dysregulation of oncogenic pathways such as PI3K/AKT. Concurrently, low-grade systemic inflammation activates IL-6/STAT3 signaling, reinforcing pro-tumorigenic transcriptional programs and epigenetic stability. This neuroendocrine–inflammatory context promotes epithelial–mesenchymal transition and cancer stem cell expansion (40, 41), while altered BDNF and NPY signaling contribute to tumor plasticity, angiogenesis, and metastatic progression (42).

Within this framework, emerging evidence further suggests that extreme physical conditions, beyond psychological stress, can modulate conserved biological programs. Studies in microgravity models, including gene expression analyses in *Drosophila melanogaster*, have identified differentially regulated genes with human homologs linked to glandular epithelial tumor biology, reinforcing the concept that extreme environmental stressors may reveal conserved oncogenic mechanisms (43).

Based on this evidence, we propose that chronic exposure to extreme stress in military contexts constitutes a systemic, hierarchical, and epigenetically reinforced pathogenic axis that contributes to the progression of glandular epithelial neoplasms. This review integrates molecular, epidemiological, and translational evidence to support this hypothesis, providing a framework for future research at the intersection of stress biology, oncology, and military medicine.

Given the integrative nature of the proposed framework, it is essential to clarify the level of evidence supporting each component and to differentiate between well-established mechanisms, emerging evidence, and more exploratory hypotheses. To improve conceptual clarity, it is important to differentiate between levels of evidence supporting the proposed framework. Certain components of the model are strongly supported by extensive experimental and clinical evidence. In particular, dysregulation of the HPA axis (including NR3C1 and FKBP5) and activation of the IL-6/STAT3 inflammatory pathway are well-established mechanisms consistently demonstrated in stress-related disorders and cancer biology (21–26, 29–32, 44–51).

In contrast, other elements of the framework, such as the involvement of BDNF and NPY in tumor-related processes, represent emerging evidence supported by growing but still incomplete translational and experimental data (33–35, 37–39, 42).

Finally, the proposed convergence between stress biology and gene signatures derived from microgravity models represents a hypothesis-generating and exploratory component of the model. This latter aspect should be interpreted as a conceptual extension aimed at identifying potential evolutionary conserved vulnerabilities, rather than as a confirmed mechanistic pathway.

This stratification of evidence is essential to properly contextualize the integrative nature of the present hypothesis and to avoid overinterpretation of preliminary findings (43) (**Supplementary Table 2**).

## 2 Materials and methods

This work is a hypothesis-driven integrative review aimed at exploring the potential role of extreme military stress as a systemic factor in the development and progression of glandular epithelial neoplasms. The study is based on a structured and comprehensive analysis of the available scientific literature (see **Supplementary Table 1**).

**Figure 1:**
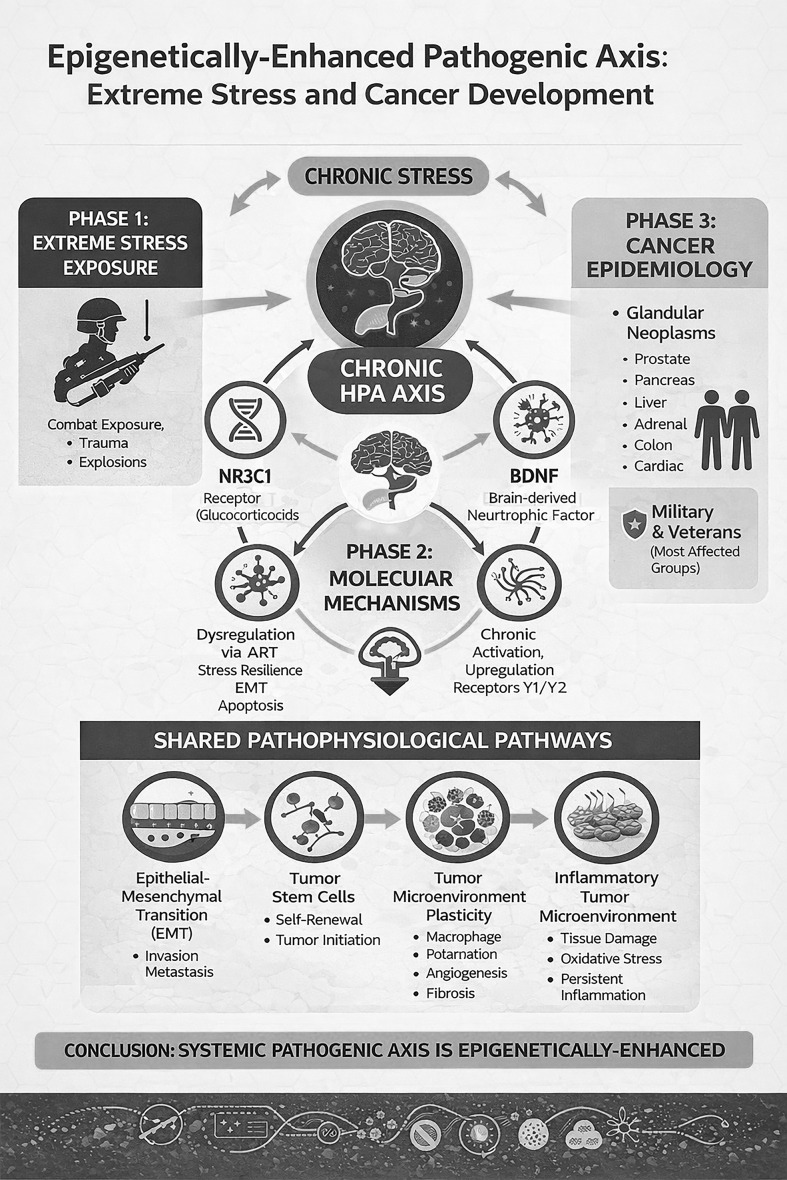
Epigenetically enhanced pathogenic axis linking extreme stress to cancer development. The schematic illustrates a three-phase model in which exposure to extreme stress drives chronic activation of the hypothalamic -pituitary -adrenal (HPA) axis, resulting in sustained glucocorticoid signaling and epigenetic remodeling of stress-responsive pathways. Phase 1 depicts the initial neuroendocrine activation. Phase 2 highlights key molecular mediators, including NR3C1 dysregulation, altered BDNF signaling, and persistent inflammatory activation, which converge to promote epithelial -mesenchymal transition (EMT), acquisition of cancer stem cell -like properties, and remodeling of the tumor microenvironment. These processes collectively facilitate tumor initiation and progression under conditions of chronic inflammation and oxidative stress. Phase 3 representes the epidemiological outcome, characterized by increased incidence of glandular malignancies in highly exposed populations, including military veterans. Overall, the model defines a stress -cancer axis driven by sustained neuroendocrine dysregulation, inflammatory signaling, and epigenetically mediated oncogenic reprogramming.

To address this hypothesis, a translational framework organized into three sequential and complementary phases was developed (see **Figure 1**). This approach was designed to conceptually reconstruct the pathogenic axis linking chronic stress exposure with oncological outcomes in military and veteran populations, using the molecular pathophysiology of post-traumatic stress disorder (PTSD) as the central integrative element.

In Phase 1, evidence on molecular and biological alterations associated with extreme stress was examined, focusing on key systems involved in stress response regulation. Particular attention was given to the hypothalamic–pituitary–adrenal (HPA) axis (NR3C1, FKBP5), neuronal plasticity and resilience mediators (BDNF, NPY), and inflammatory signaling pathways (IL-6), with the aim of identifying interconnected mechanisms within an allostatic network.

In Phase 2, epidemiological and clinical evidence on cancer burden in military and veteran populations was analyzed, with emphasis on recent large-scale cohort studies. This phase enabled the identification of glandular epithelial neoplasms potentially susceptible to neuroimmunoendocrine dysregulation, including hepatocellular carcinoma, pancreatic ductal adenocarcinoma, and prostate cancer.

In Phase 3, findings from the previous phases were integrated to develop a unified mechanistic hypothesis. This synthesis explored how stress-related molecular alterations may converge on key oncogenic pathways, including PI3K/AKT, IL-6/STAT3, and BDNF/TrkB signaling, as well as processes such as epithelial–mesenchymal transition and cancer stem cell expansion. Based on this integration, a hierarchical model of stress-associated tumor progression was proposed.

This conceptual framework supports the formulation of a testable hypothesis in which chronic military stress acts as a systemic and underrecognized driver of oncogenesis.

### 2.1 Construction of ordinal indices

To complement the conceptual analysis, a semi-quantitative integrative approach was applied. Recent literature (2024–2026) on cancer prevalence in military populations was reviewed, prioritizing epidemiological studies, institutional cohorts, and meta-analyses.

Based on consistency across studies, eight recurrent cancer types were identified. A Relative Frequency Index (3 = high, 2 = moderate, 1 = low) was assigned according to recurrence, cohort size, and epidemiological relevance. Additionally, a Military Exposure Association Index was developed to estimate the strength of association between each neoplasm and military-related exposures, including environmental and stress-related factors.

This approach enabled the identification of convergent patterns and supported the generation of mechanistic hypotheses, without implying causal inference.

### 2.2 Search strategy

Relevant literature was identified through searches in Scopus, PubMed, Web of Science, and Google Scholar. These databases were selected for their broad multidisciplinary coverage and inclusion of peer-reviewed studies.

The search strategy was guided by the conceptual framework described above and structured to capture evidence related to:

1. molecular effects of extreme stress,
2. cancer epidemiology in military populations, and.
3. mechanistic links between stress mediators and oncogenic pathways.

Search equations included combinations of terms related to military populations, stress exposure (including PTSD), target genes (FKBP5, NR3C1, BDNF, IL6, SLC6A4, NPY), and cancer-related processes. Representative search strategies are provided below:

1. TITLE-ABS-KEY ((military OR soldier* OR combat OR “armed forces”) AND (“extreme stress” OR “combat stress” OR “operational stress” OR “psychological stress” OR PTSD) AND (FKBP5 OR NR3C1 OR BDNF OR IL6 OR SLC6A4 OR NPY)).
2. TITLE-ABS-KEY (cancer) AND TITLE-ABS-KEY (veterans OR “military personnel”) AND TITLE-ABS-KEY (prevalence OR incidence OR epidemiology).
3. (“Neoplasms, Glandular and Epithelial”[Mesh]) AND (NR3C1[tiab] OR “Glucocorticoid Receptor”[tiab] OR GR[tiab] OR FKBP5[tiab] OR BDNF[tiab] OR “Brain-Derived Neurotrophic Factor”[tiab] OR NPY[tiab] OR “Neuropeptide Y”[tiab] OR IL6[tiab] OR IL-6[tiab] OR “Interleukin-6”[tiab]).
4. (NR3C1[tiab] OR FKBP5[tiab] OR IL6[tiab] OR IL-6[tiab] OR BDNF[tiab] OR NPY[tiab]) AND (PI3K[tiab] OR AKT[tiab] OR “PI3K/AKT”[tiab] OR STAT3[tiab] OR EMT[tiab] OR “epithelial mesenchymal transition”[tiab] OR “cancer stem cells”[tiab] OR stemness[tiab] OR angiogenesis[tiab]).

### 2.3 Study selection and categorization

Eligible studies included original research articles, systematic reviews, and meta-analyses published in peer-reviewed journals with full-text availability. Studies involving military populations, veterans, or relevant experimental models were prioritized.

Selection was based on relevance to the proposed stress–oncogenesis framework. Articles were subsequently categorized according to their primary contribution as molecular, epidemiological, or mechanistic evidence, in alignment with the three-phase conceptual model.

**Figure 2:**
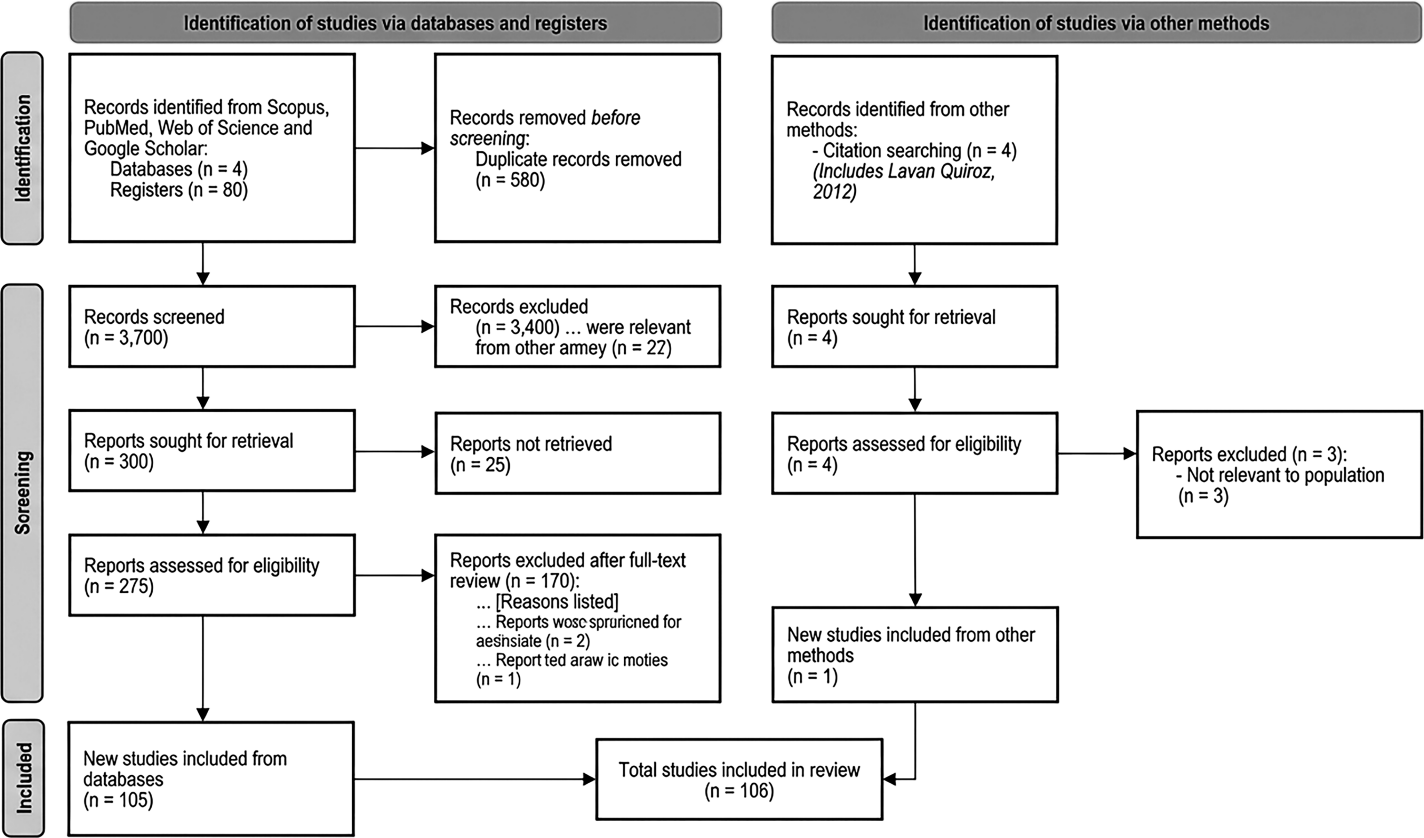
PRISMA flow diagram. The diagram illustrates the systematic process of identification, screening, eligibility assessment, and final inclusion of studies. It integrates records retrieved from scientific databases and additional sources, detailing the exclusion and selection stages utilized throughout the review.

A total of 106 studies were selected and included. The study selection process followed the PRISMA flow diagram, detailing the number of records identified, screened, excluded, and ultimately included in the analysis (see **Figure 2**).

### 2.4 Inclusion and exclusion criteria

Inclusion criteria comprised studies addressing at least one of the following domains: molecular alterations associated with extreme stress, epidemiological evidence of cancer in military populations, or mechanistic links between stress-related mediators and oncogenic pathways.

Exclusion criteria included studies lacking relevance to the stress–oncogenesis interaction, those restricted to civilian populations without translational applicability, duplicate records, and publications without full-text access or adequate methodological quality.

Although the proposed framework may apply broadly, the analysis focused on glandular epithelial tumors due to their biological plausibility and epidemiological relevance.

### 2.5 Data extraction and analysis

Data were extracted using a structured approach, including study characteristics, population type, biomarkers evaluated, oncological outcomes, and key findings. The objective was to identify consistent patterns and mechanistic links supporting the proposed hypothesis.

Protein interaction and signaling pathway relationships were explored using the STRING database (32), enabling further integration of molecular evidence.

### 2.6 Ethical considerations

This study is based exclusively on previously published data. All included studies complied with established ethical standards for research involving human subjects or experimental models.

## 3 Results

### 3.1 Neuroendocrine and immuno-inflammatory dysregulation induced by extreme military stress: molecular integration of NR3C1, FKBP5, BDNF, NPY, and IL-6 in PTSD

**Table 1** synthesizes the available evidence on five central molecular nodes involved in the biological response to chronic stress in military and veteran populations. For each molecule, the table integrates functional alterations observed in PTSD, the underlying biological mechanisms, supporting genetic and epigenetic evidence, associated clinical outcomes, and their interactions within a neuroimmunoendocrine network.

**Table 1:** Molecular dysregulation associated with extreme military stress in PTSD.

Molecule / gene	Alteration in military PTSD	Biological mechanism involved	Genetic / epigenetic evidence	Associated clinical consequences	Interactions within the network	References
NR3C1 (Glucocorticoid Receptor)	GR hypersensitivity; low baseline cortisol levels with increased reactivity	Exacerbated negative feedback of the HPA axis	Promoter 1F hypomethylation; 9β polymorphism	Vulnerability to PTSD; altered treatment response	Direct interaction with FKBP5; regulates IL6	Yehuda et al. (44); Yehuda et al. (48); van Zuiden et al. (45); Castro-Vale et al. (46); Yehuda et al. (47)
FKBP5	Chronic overexpression in carriers of risk alleles	Decreases GR affinity for cortisol; relative glucocorticoid resistance	Post-trauma intronic demethylation; rs1360780	Prolonged stress response; higher PTSD risk	Modulates NR3C1; affects inflammatory regulation	Kang et al. (49); Young et al. (50); Watkins et al. (52); Lee et al. (53); Bishop et al. (54)
BDNF	Functional decrease; variable plasma alterations	Reduced neuronal plasticity; hippocampal-amygdala-PFC impairment	Val66Met polymorphism; promoter hypermethylation	Cognitive deficit; suicide risk; poorer therapeutic response	Inhibited by IL6; modulator of resilience	Bruenig et al. (55); Dretsch et al. (56); Zhang et al. (57); Kim et al. (36); Rippey et al. (58); Sher et al. (59); Wu et al. (60); Zalta et al. (61)
NPY	Chronic low levels in plasma and CSF	Decreased endogenous anxiolytic effect; sympathetic hyperactivation	Altered mobilization under stress	Greater PTSD severity; suicide risk; cardiometabolic comorbidity	Inhibits HPA axis; modulates sympathetic system	Morgan et al. (62); Yehuda et al. (63); Sah et al. (64, 65); Rasmusson et al., (66); Reijnen et al. (67); Sher et al. (68); Rasmusson (69)
IL6	Chronic circulating elevation	Persistent proinflammatory state; tissue GR resistance	Associated with PTSD severity	Insulin resistance; chronic pain; cognitive impairment	Inhibits BDNF; interacts with HPA axis	Rodney et al. (70); Kanefsky et al. (71); Gill et al. (72); Brooks-Holliday et al. (73); Bruenig et al. (27); Bersani et al. (28); Somvanshi et al. (74); Breen et al. (75)

Rather than representing isolated findings, the data show a convergent and coordinated pattern of dysregulation. NR3C1, which encodes the glucocorticoid receptor, consistently exhibits increased sensitivity associated with promoter hypomethylation and specific risk polymorphisms, suggesting a persistent alteration in hypothalamic–pituitary–adrenal (HPA) axis regulation and heightened vulnerability to post-traumatic stress disorder (PTSD) (44–48). This alteration does not occur in isolation but is functionally reinforced by FKBP5, a key regulator of glucocorticoid signaling, which shows chronic overexpression, particularly in carriers of the rs1360780 allele. This overexpression, driven by trauma-induced demethylation, leads to glucocorticoid resistance and prolonged cortisol signaling (49, 50, 52–54). Together, NR3C1 and FKBP5 disrupt the normal negative feedback of the HPA axis, sustaining stress activation.

This neuroendocrine disruption is not limited to hormonal regulation but also directly impacts other key biological systems. In this regard, chronic stress affects neuronal plasticity through BDNF. Evidence consistently shows reduced BDNF activity, associated with both the Val66Met polymorphism and promoter hypermethylation. This reduction is linked to cognitive deficits, increased suicide risk, and poorer treatment response (36, 55–61).

In parallel, NPY levels are chronically reduced in both plasma and cerebrospinal fluid in veterans with PTSD. This depletion removes a key endogenous anxiolytic mechanism, contributing to sustained sympathetic activation, greater symptom severity, and increased cardiometabolic risk (62–69).

Finally, IL-6 remains persistently elevated, reflecting a state of chronic low-grade inflammation that develops in parallel with neuroendocrine dysregulation. Elevated IL-6 levels are associated with insulin resistance, chronic pain, and cognitive impairment, particularly in the context of tissue-level glucocorticoid resistance. (27, 28, 70–75).

Taken together, these findings allow the integration of this inflammatory component into a broader model in which these five molecules form a hierarchical and interconnected network. Their sustained dysregulation under conditions of extreme stress extends beyond neuropsychiatric manifestations and may contribute to systemic comorbidities, including glandular epithelial neoplasms (see **Table 1**).

**Table 2** synthesizes the main systemic comorbidities observed in veterans exposed to extreme stress, organized by clinical domain and linked to the molecular alterations described above. In the neurocognitive domain, reduced BDNF and elevated IL-6 converge to produce a state of chronic inflammation and impaired neuroplasticity. This combination explains the deficits in memory and executive function consistently reported in PTSD populations (58, 76–78).

**Table 2:** Systemic consequences derived from molecular dysregulation.

Clinical domain	Molecules involved	Predominant mechanism	Manifestation in veterans	References
Neurocognitive	BDNF, IL6	Inflammation + reduced plasticity	Memory deficit, executive dysfunction	Rippey et al. (58); Domitrovic Spudic et al. (76); Guo et al. (77); Smith et al. (78)
Suicide	NPY, BDNF, FKBP5, NR3C1	HPA axis dysfunction + neuronal vulnerability	Suicidal ideation and attempts	Sher et al. (59); Sher et al. (68); Zhang et al. (79); Boscarino et al. (80)
Metabolic	IL6, NR3C1, FKBP5	Inflammation + dysfunctional cortisol	Metabolic syndrome	Blessing et al. (81); Chacko et al. (3)
Chronic pain	IL6, HPA axis	Inflammatory sensitization	Persistent pain post-TBI	Lerman et al. (82); Kanefsky et al. (71)
Cardiovascular	NPY, IL6	Sympathetic hyperactivation + inflammation	Increased cardiovascular risk	Rasmusson (69); Ciumaşu-Rîmbu et al. (83)

At a higher level of clinical complexity, suicidal behavior emerges from a more intricate molecular interplay involving dysregulation of the hypothalamic–pituitary–adrenal (HPA) axis, reduced levels of NPY and BDNF, and the presence of risk variants in FKBP5 and NR3C1. Together, these alterations create a state of increased neuronal vulnerability that predisposes to suicidal ideation and attempts (59, 68, 79, 80).

Complementarily, these same molecular alterations are not confined to the neuropsychiatric domain but also have systemic repercussions. At the metabolic level, persistent elevation of IL-6 and altered glucocorticoid signaling mediated by NR3C1 and FKBP5 contribute to the development of metabolic syndrome, a condition highly prevalent among veterans. (3, 81).

In parallel, chronic pain, particularly following mild traumatic brain injury, is sustained by mechanisms of inflammatory sensitization. In this context, IL-6 and dysregulation of the HPA axis play a central role. (71, 82).

Consistently, cardiovascular risk is also increased, driven by the combination of chronic inflammation (IL-6) and sustained sympathetic activation associated with NPY depletion. (69, 83).

This finding integrates into a broader pattern of systemic comorbidity. Overall, **Table 2** highlights that the same molecular nodes involved in stress regulation also underlie multiple systemic comorbidities. This supports a unified biological framework and reinforces the need for integrated translational approaches in veteran populations (see **Table 2**).

**Table 3** integrates these findings into a hierarchical model of stress-related pathophysiology. The model organizes the evidence into four components: the affected molecular node, the primary alteration, the resulting biological cascade, and the associated clinical outcomes. In this context, the hypothalamic–pituitary–adrenal (HPA) axis, represented by NR3C1 and FKBP5, emerges as the central component of the model, exhibiting impaired negative feedback. This leads to abnormal cortisol dynamics and glucocorticoid resistance, increasing vulnerability to post-traumatic stress disorder (PTSD) (44, 49, 50). BDNF shows reduced secretion and epigenetic dysregulation, which translate into impaired fear extinction and cognitive deficits (36, 55, 58). In parallel, NPY undergoes sustained depletion, removing a key anxiolytic mechanism and promoting persistent sympathetic activation and anxiety states (62, 63, 68). Similarly, IL-6 remains chronically elevated, driving systemic inflammation and contributing to metabolic disorders such as insulin resistance and metabolic syndrome. (27, 28, 81).

**Table 3:** Integrated model of allostatic dysregulation in extreme military stress.

Central node	Primary disruption	Pathophysiological cascade	Systemic outcome	References
HPA axis (NR3C1/FKBP5)	Altered feedback	Dysfunctional cortisol + tissue resistance	PTSD vulnerability	Yehuda et al. (44); Kang et al. (49); Young et al. (50)
Neuronal plasticity (BDNF)	Reduced secretion / altered epigenetics	Impaired fear extinction	Cognitive decline	Bruenig et al. (55); Kim et al. (36); Rippey et al. (58)
Anxiolytic modulation (NPY)	Chronic depletion	Sympathetic hyperactivation	Persistent anxiety	Morgan et al. (62); Yehuda et al. (63); Sher et al. (68)
Inflammation (IL6)	Sustained elevation	Systemic neuroinflammation	Metabolic comorbidity	Bruenig et al. (27); Bersani et al. (28); Blessing et al. (81)

In summary, this model proposes that these five nodes function as an integrated allostatic network. Their chronic dysregulation under extreme stress conditions generates a vulnerability phenotype that extends beyond psychiatric disease and may contribute to systemic disorders, including glandular epithelial neoplasms (see **Table 3**).

### 3.2 Oncologic burden in military personnel and veterans: epidemiological integration and molecular characterization

#### 3.2.1 Most frequent cancers in military personnel and veterans: recent evidence from the military and veteran health systems

An analysis of recent literature (2024–2026) (see **Table 4**) reveals a consistent pattern of neoplasms affecting military personnel and veterans. Rather than representing isolated findings, the available evidence converges toward a defined epidemiological profile. **Table 4** synthesizes this information by organizing tumor types according to their relative frequency, clinical relevance, and supporting data from major systems such as the Veterans Health Administration (VHA), the Military Health System (MHS), and the Million Veteran Program (MVP).

**Table 4:** Comparative overview of frequent cancers in military personnel / veterans.

Cancer type	Evidence of frequency/prevalence in military/veterans	Relevant indicators	Representative studies
Prostate cancer	One of the most frequent in male veterans; high incidence within the VA system.	High incidence; focus on screening, progression, racial disparities.	Candelieri-Surette et al. (5); Green-Lott et al. (85); Kronstedt et al. (86)
Lung / bronchial cancer	Frequently reported in veterans; impact of LDCT screening demonstrated.	High prevalence associated with tobacco and environmental exposures.	Edwards et al. (6); Brandt et al. (7); Lewis et al. (2025)
Colorectal cancer	High burden in veteran population; screening and diagnoses within VA documented.	Associated with socioeconomic inequalities, emergencies, and survival.	Beydoun et al. (8); Englum et al. (9); Khalaf et al. (10)
Skin melanoma	Relevant frequency, related to intense sun exposure.	Recorded in Veterans Health data; definition issues in EHR.	Wheless et al. (11); Rezaei et al. (13); Chang et al. (12)
Testicular tumors	Relevant in young male veterans; documented comorbidities.	Associations with metabolic syndrome and long-term sequelae.	Puri et al. (14); Yodkhunnatham et al. (87)
Breast cancer	More frequent in female veterans; analysis of disparities and access.	Evaluation of surgery, genetics, and demographic characteristics.	Eaglehouse et al. (15); Li et al. (16); Zullig et al. (17)
Cervical cancer	Less extensive data, but active screening in military system.	Evidence in studies combined with breast screening.	Segel et al. (18)
Thyroid cancer	Reported in military cohorts; possible link to environmental exposures.	Studies of racial patterns and preliminary epigenetic data.	Eaglehouse et al. (19); Amreen et al. (20)

In this context, prostate cancer emerges as the most prevalent solid malignancy among male veterans. Multiple studies consistently report high incidence rates within the VA system, also addressing key dimensions such as metastatic castration-resistant disease (mCRPC) (5), the protective role of healthy lifestyle factors (84), and cohort-specific outcomes in Vietnam-era veterans (85). In line with these findings, recent systematic reviews suggest that veterans may constitute a high-risk population, potentially warranting intensified screening strategies (86).

Similarly, lung and bronchial cancer also represent a significant oncologic burden. Their high incidence is strongly associated with historical tobacco use and occupational exposures. Importantly, the implementation of low-dose computed tomography (LDCT) screening has shifted diagnosis toward earlier stages and reduced mortality in veteran populations (6). Complementary surgical studies further identify prognostic factors associated with improved survival in non-small cell lung cancer (NSCLC) (7).

Along the same lines, colorectal cancer remains highly prevalent, particularly in aging cohorts. Current evidence highlights the role of social determinants, such as housing status, in screening access, incidence, and mortality (8). Additionally, disruptions during the COVID-19 pandemic have highlighted diagnostic inequalities and delays in care (9), while analyses of emergency presentations provide insight into disease progression and gaps within the healthcare system (10).

Complementarily, cutaneous melanoma illustrates the impact of environmental and occupational factors in this population. It is particularly relevant due to cumulative ultraviolet radiation exposure during military training and deployments. Data from the Million Veteran Program highlight methodological limitations in case identification within electronic health records. (11), whereas broader analyses underscore the overall burden of skin cancer and the need for improved prevention and dermatological access (12, 13).

In this context, other less frequent tumors also carry specific clinical relevance. Testicular cancer, although less common, is of particular importance in younger populations. Evidence from the VA system highlights the long-term burden of metabolic syndrome and other comorbidities among survivors. (14).

Among female veterans, breast cancer is the most common malignancy. Research has identified disparities in surgical outcomes across racial and ethnic groups (15), as well as gaps in access to germline genetic testing (16). In parallel, cervical cancer studies point to variability in screening adherence driven by geographic and individual factors (18).

Finally, thyroid cancer has been examined in relation to treatment disparities and potential service-related exposures. Notably, emerging studies suggest epigenetic alterations associated with Agent Orange exposure, pointing to possible mechanistic links between environmental exposure and tumor biology (20).

Collectively, these findings demonstrate that prostate, lung, colorectal, melanoma, testicular, breast, cervical, and thyroid cancers constitute the predominant oncological burden in military and veteran populations. Importantly, their distribution reflects the combined influence of occupational exposures, behavioral risk factors, and social determinants of health, underscoring the need for tailored prevention and screening strategies (see **Table 4**).

#### 3.2.2 Ordinal epidemiological profile of oncological burden and military exposure

**Figure 3:**
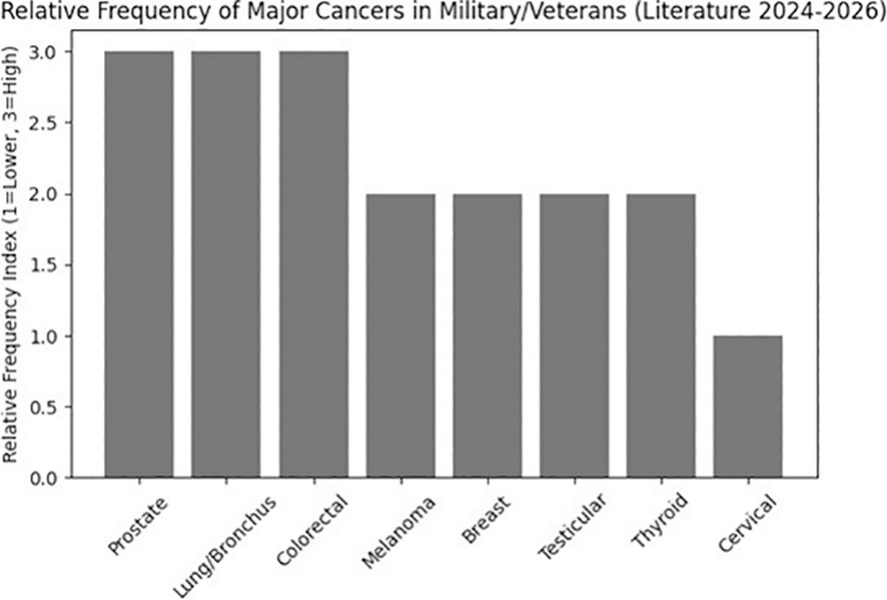
Relative frequency of primary cancer types in military personnel and veterans (2024 -2026). The ordinal scale categorizes relative frequency based on recent literature as follows: 1, low frequency; 2, moderate frequency; and 3, high frequency. The chart shows that prostate, lung/bronchial, and colorectal cancers form the core group of highest-frequency malignancies in military and veteran populations. These cancers represent the greatest oncological burden, particularly in aging cohorts, likely reflecting the combined effects of demographic factors, cumulative environmental exposures, and chronic inflammatory processes. Melanoma, breast, testicular, and thyroid cancers are observed at intermediate frequencies, whereas cervical cancer consistently shows the lowest relative frequency in this population.

**Figure 4:**
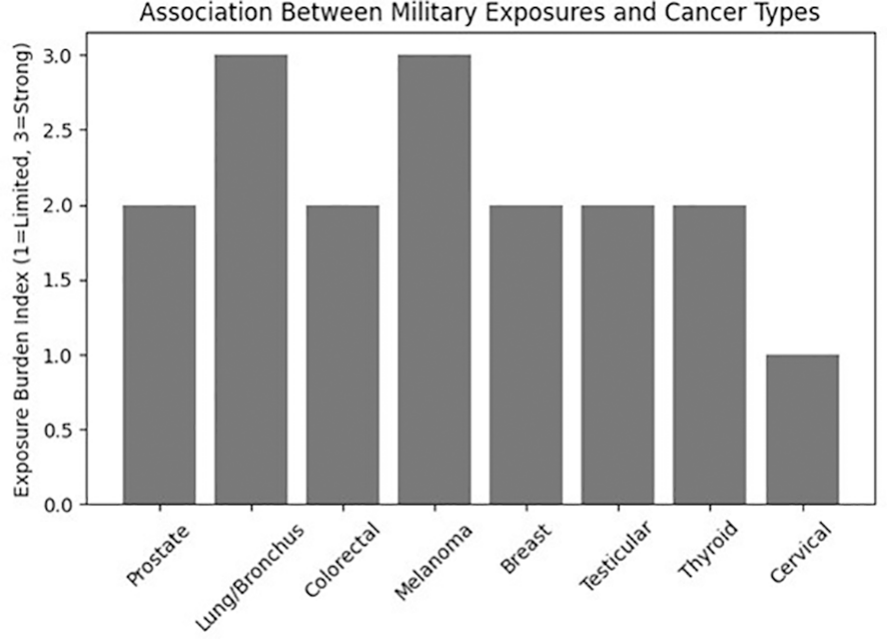
ssociation between ccancer types and military exposure burden. The ordinal scale represents the estimated degree of association between military occupational exposures and oncological risk: 1, limited association; 2, moderate association; 3, strong association. Lung cancer and melanoma show the strongest associations with military-specific exposures, including hydrocarbon combustion, ultraviolet radiation, and environmental toxins. Other cancer types demonstrate moderate associations, potentially mediated by systemic mechanisms such as chronic inflammation, endocrine dysfunction, or exposure to solvents and endocrine-disrupting compounds. In contrast, cervical cancer shows a limited association, consistent with its predominantly viral etiology.

The comparative ordinal analysis reveals a structured distribution of oncological burden (see **Figures 3**, **4**), allowing for clearer interpretation of how frequency and exposure interact across tumor types.

Based on the Relative Frequency Index, prostate, lung/bronchial, and colorectal cancers form a high-frequency group (score = 3), representing the epidemiological core of malignancies in these populations. A second tier (score = 2) includes melanoma, breast cancer, testicular tumors, and thyroid cancer, while cervical cancer consistently appears as low frequency (score = 1).

When analyzed through the Military Exposure Association Index, a distinct pattern emerges. Lung cancer and melanoma show the strongest association with military exposures (score = 3), indicating a clear occupational component. In contrast, prostate, colorectal, breast, testicular, and thyroid cancers demonstrate moderate associations (score = 2), suggesting multifactorial etiologies. Cervical cancer again shows minimal association (score = 1).

A key insight from this comparison is the dissociation between frequency and exposure. Lung cancer uniquely combines both high frequency and strong exposure linkage, positioning it as the tumor with the greatest composite burden. Conversely, prostate cancer is highly prevalent but only moderately associated with exposure, while melanoma shows the opposite pattern.

Overall, this analysis supports the existence of an epidemiological gradient that differentiates tumors primarily driven by cumulative systemic factors from those more directly linked to occupational exposures.

#### 3.2.3 Molecular contextualization of epidemiological gradients

The observed epidemiological patterns align with emerging molecular frameworks that connect chronic stress biology with cancer susceptibility.

Tumors within the high-frequency group, particularly prostate and colorectal cancers, are characterized by sensitivity to systemic endocrine and inflammatory signals. This is consistent with chronic activation of the HPA axis and sustained NR3C1-mediated glucocorticoid signaling, processes known to influence metabolism, immune regulation, and epithelial cell dynamics.

Similarly, cancers with strong exposure associations overlap with pathways influenced by stress-related molecular regulators. Dysregulation of FKBP5 can prolong cortisol signaling and alter key oncogenic pathways such as PI3K/AKT, potentially promoting tumor survival and resistance. In parallel, chronic IL-6 elevation provides a pro-inflammatory environment that may facilitate tumor initiation and progression.

Within this framework, cancers like lung malignancies represent a convergence point between environmental exposure and stress-mediated biological vulnerability. In contrast, tumors with high frequency but weaker exposure links may reflect cumulative neuroendocrine and immunometabolic dysregulation rather than direct carcinogenic insults.

Taken together, these findings support the concept of a stress–inflammation–oncogenesis axis, in which chronic neuroendocrine dysregulation acts as a biological amplifier of cancer risk in military populations.

#### 3.2.4 Summary of oncological burden (2024–2026)

**Table 5** presents a comparative synthesis of the most frequently reported cancer types in military and veteran populations, based on epidemiological literature published between 2024 and 2026. This classification allows visualization of both the distribution of oncological burden and the key clinical and epidemiological indicators associated with each neoplasm.

**Table 5:** Comparison of the most frequent cancer types in military personnel and veterans according to recent literature (2024–2026).

Cancer type	Frequency evidence	Key indicators
Prostate	High	Screening, progression, disparities
Lung/Bronchial	High	LDCT screening, mortality
Colorectal	Medium-High	Socioeconomic inequalities
Skin melanoma	Medium	Sun exposure, incidence
Testicular	Medium	Chronic sequelae
Breast	Medium	Disparities, genetics
Cervical	Low-Medium	Integrated screening
Thyroid	Low-Medium	Environmental exposures

In this context, prostate and lung/bronchial cancers consistently emerge as the most frequent malignancies in this population. In prostate cancer, the evidence emphasizes screening strategies, disease progression patterns, and persistent racial disparities (5, 85, 86). In parallel, lung cancer prevalence is strongly linked to occupational exposures and smoking history, with multiple studies supporting the impact of low-dose CT screening on early detection and mortality reduction (6, 7).

A second group, with medium-to-high frequency, includes colorectal cancer, cutaneous melanoma, and testicular tumors. Colorectal cancer shows a clear association with socioeconomic inequalities, as well as with delayed diagnosis and emergency presentations (8–10). Melanoma is primarily linked to cumulative solar exposure during training and deployment (11, 13), while testicular tumors are notable for their long-term metabolic and bone-related sequelae in young veterans (14).

Among female veterans, breast cancer represents the most relevant neoplasm. Current evidence highlights disparities in outcomes and differences in access to genetic testing and specialized care (15–17). Cervical cancer and thyroid cancer complete the spectrum with medium-to-low frequency. Cervical cancer is mainly associated with variability in screening adherence (18–20), whereas thyroid cancer has been linked to environmental exposures such as Agent Orange, with emerging evidence suggesting potential epigenetic mechanisms (19, 20).

Overall, this epidemiological profile shows a structured distribution of cancer burden, where high-frequency tumors coexist with others more closely associated with specific exposures or demographic factors. This characterization provides the basis for exploring, in subsequent sections, how extreme stress-induced neuroendocrine dysregulation may contribute to the progression of glandular epithelial neoplasms (see **Table 5**).

### 3.3 Most frequent cancers in military personnel and veterans: extreme stress, HPA axis, and glandular epithelial tumors

**Table 6** (see **Table 6**) synthesizes the biological axes, molecular mechanisms, and functional evidence supporting an integrative model linking chronic stress-induced dysregulation with tumor progression in hepatocellular carcinoma (HCC), pancreatic ductal adenocarcinoma (PDAC), and prostate cancer.

**Table 6:** Neuroimmunoendocrine-epigenetic model associated with tumor progression in glandular epithelial neoplasias under prolonged extreme stress.

Biological axis	Mecanismo molecular	Reported functional evidence	Relevance in HCC	Relevance in PDAC	Relevance in prostate cancer	Implication in chronic military stress
HPA – NR3C1	Chronic glucocorticoid activation; NR3C1 acts as ligand-dependent transcription factor; epigenetic remodeling in glucocorticoid-sensitive promoters	GR signaling disruption favors steatosis and progression to HCC in murine models (Mueller et al. (21)	Altered hepatic homeostasis and hepatocarcinogenic progression	Tumor metabolic modulation	Influence on hormone-dependent tissues	Sustained hypercortisolemia induces persistent GR activation
NR3C1 – BAG-1	Antiapoptotic modulation and GR complex stability	BAG-1 regulates antiapoptotic signals associated with GR (Zhou et al. (22)	Clonal survival advantage	Cellular resistance	Tumor survival	Selection of resistant clones under chronic signaling
FKBP5 – AKT	Immunophilin regulating GR sensitivity; negative modulation of AKT	Regulates chemotherapy response via AKT (Pei et al. (25); involvement in tumor etiology and chemoresistance Li et al. (51); radiation-induced apoptosis (Romano et al., 2010)	Altered proliferation/apoptosis balance	Relevant in constitutive AKT activation and therapeutic resistance	Hormone-dependent tumor sensitive to GR modulation	Stress-induced overexpression
IL-6 / STAT3	JAK/STAT3 activation; metabolic and epigenetic reprogramming; autocrine inflammatory loops	STAT3 activation by IL-6 Wegenka et al. (29); Zhang et al. (30); metabolic reprogramming in HCC (Wang et al. (31); induction of metastatic stem-like phenotype Mitra et al. (32); IL-6 correlation with tumor proliferation Yamaji et al. (88)	Hepatocarcinogenesis and progression	Pancreatic tumorigenesis	Inflammatory proliferative activation	Low-grade systemic inflammation associated with chronic stress
BDNF / TrkB	RAS/MAPK activation; cellular plasticity; therapeutic resistance	MAPK activation via TrkB Easton et al. (33); TrkB inhibition reduces tumor growth and potentiates chemotherapy Croucher et al. (34),; Iyer et al. (35)	Potential contribution to tumor survival	Tumor plasticity and resistance	Participation in epithelial survival	Systemic BDNF alteration under extreme stress
NPY – Y1/Y2	Vascular tone regulation; angiogenesis; microvascular remodeling	Y1/Y2 receptors described Sheikh et al. (37); vasoconstrictor effects Tschöpl et al. (38); high-affinity antagonists available Daniels et al. (39)	Tumor angiogenesis in HCC	Tumor vascular support	Relevant in angiogenic tumors	Sustained sympathetic activation in operational stress
Integrative HPA–IL6–Epigenetics model	Hypercortisolemia → NR3C1 → FKBP5 → AKT; IL-6/STAT3 inflammation; epigenetic reprogramming; EMT and cancer stem cell expansion	FKBP5 regulation and stress epigenetics Binder (23); Zannas et al. (24); cancer inflammatory axis Grivennikov & Karin (89); Johnson et al. (90); EMT and epigenetics Thiery et al. (40); Easwaran et al. (41); tumor plasticity (Desmet & Peeper, 2021)	Aggressive HCC progression	PDAC progression and resistance	Prostate metastatic phenotype	Extreme military stress as systemic pathogenic axis

Within this framework, at the neuroendocrine level, the HPA–NR3C1 axis constitutes the central regulatory framework. Persistent activation of the glucocorticoid receptor promotes epigenetic remodeling and metabolic alterations that may favor tumor development (21). Within this axis, the interaction between NR3C1 and BAG-1 introduces an anti-apoptotic component that enhances cellular survival under sustained glucocorticoid signaling (22).

In continuity with this system, the FKBP5–AKT axis links glucocorticoid sensitivity with key oncogenic signaling pathways. Dysregulation of FKBP5 affects PI3K/AKT pathway activity, influencing cell proliferation, survival, and therapeutic resistance (25, 26, 51). This mechanism is particularly relevant in tumors characterized by high proliferative activity and resistance to treatment.

Complementarily, the IL-6/STAT3 axis represents the inflammatory component of the model. Chronic activation of this pathway promotes metabolic reprogramming, epithelial–mesenchymal transition (EMT), and the acquisition of cancer stem cell (CSC) phenotypes (29–32, 88). This axis provides a mechanistic link between systemic inflammation and tumor progression.

In this context, other axes such as BDNF/TrkB and NPY–Y1/Y2 further expand this biological framework. BDNF signaling contributes to cellular plasticity and survival through activation of the MAPK pathway, while NPY influences vascular remodeling and angiogenesis within the tumor microenvironment. (33–35, 37–39).

The final integration of these axes suggests that hypercortisolemia, chronic inflammation, and epigenetic reprogramming converge to promote EMT and CSC expansion. Together, these processes define a system-level pathogenic mechanism potentially activated by extreme stress in military and veteran populations (23, 24, 40–42, 89, 90).

## 4 Discussion

### 4.1 Neuroendocrine and immuno-inflammatory dysregulation induced by extreme military stress: molecular integration of NR3C1, FKBP5, BDNF, NPY, and IL-6 in PTSD

Military service exposes individuals to sustained and extreme psychological and physical stress, placing a significant burden on the systems that maintain physiological homeostasis (1). Although many individuals adapt successfully, a considerable proportion develop chronic disorders. PTSD is the most representative condition, with a high prevalence among combat veterans (2). Importantly, PTSD should not be considered an isolated psychiatric entity. Instead, it represents a systemic disorder involving coordinated alterations in neuroendocrine, immune, and autonomic systems (3, 4).

In line with the results presented in Phase 1, current evidence supports a shift from purely psychological models toward integrated biological frameworks. These frameworks emphasize the interaction between genetic susceptibility, traumatic exposure, and environmentally driven epigenetic modifications (91). Within this context, five molecular components NR3C1, FKBP5, BDNF, NPY, and IL-6 emerge as central and interconnected regulators of the stress response.

#### 4.1.1 The HPA axis at the center of the storm: NR3C1 and FKBP5

**Figure 5:**
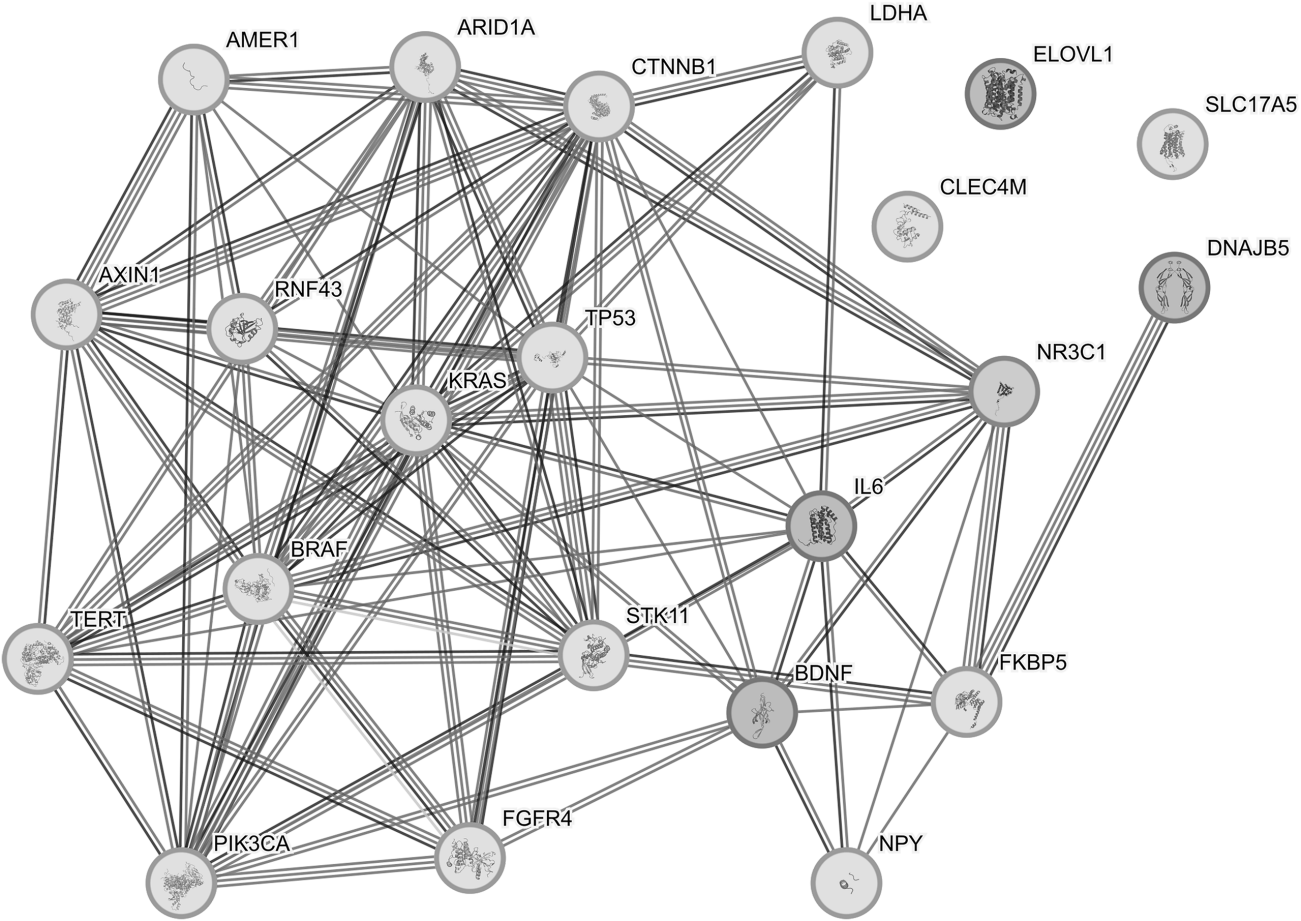
Network of stress-sensitive genes involved in neuroendocrine, inflammatory, redox, and proliferative regulation. The image illustrates an integrated gene network modulated by chronic stress and glucocorticoid signaling, organized around NR3C1 (glucocorticoid receptor) as the central regulatory hub. NR3C1 orchestrates transcriptional programs involved in inflammation, metabolism, and cell proliferation, and its activity is finely regulated by the co-chaperones FKBP5 and FKBP4, which modulate receptor sensitivity and intracellular trafficking. At the endocrine regulatory level, NR3C2 and HSD11B2 fine-tune glucocorticoid and mineralocorticoid availability and activity, thereby maintaining hormonal homeostasis, while CRHBP regulates corticotropin-releasing hormone signaling and TSC22D3 mediates glucocorticoid-dependent anti-inflammatory responses. In parallel, redox homeostasis is sustained by GSR and MT1B, which support antioxidant defense and metal detoxification, together with GGACT, which participates in γ-glutamyl cycle metabolism. Finally, GRIP1 and SKA2 link intracellular signaling pathways to vesicular trafficking and chromosomal stability, integrating stress signaling with cellular maintenance mechanisms. Collectively, this hierarchical network highlights how chronic stress drives coordinated neuroendocrine, inflammatory, redox, and proliferative reprogramming through a central glucocorticoid-regulated axis.

The hypothalamic–pituitary–adrenal (HPA) axis is the primary system coordinating the physiological response to stress. Its activation leads to the release of cortisol, which acts through the glucocorticoid receptor encoded by NR3C1. This receptor regulates stress-related gene expression and mediates the negative feedback that terminates the response. However, as shown in the results, this system is persistently altered in post-traumatic stress disorder (PTSD), as hypomethylation of the NR3C1 promoter region has been consistently observed in veterans with PTSD (48) an epigenetic change associated with increased receptor expression and consistent with findings of elevated glucocorticoid receptor levels in lymphocytes (45). Functionally, this may explain the characteristic pattern of enhanced feedback sensitivity alongside low basal cortisol levels (44). In this context, FKBP5 acts as a key modulator of the same system, normally providing short-term regulation of glucocorticoid receptor activity (see **Figure 5**); however, in individuals carrying risk variants such as rs1360780, trauma induces persistent demethylation and chronic overexpression of FKBP5 (49), leading to glucocorticoid resistance and impaired feedback control, thereby prolonging stress activation (50, 52).

Together, these findings indicate that NR3C1 and FKBP5 do not act independently. Instead, they form a tightly coupled regulatory unit whose dysregulation sustains HPA axis dysfunction, as observed in Phase 1.

#### 4.1.2 Neuronal plasticity and resilience: the role of BDNF

The results also highlight the role of BDNF as a key mediator of neuronal plasticity. Reduced BDNF activity, associated with both genetic (Val66Met) and epigenetic mechanisms, contributes to impaired cognitive resilience (36, 55–61, 92, 93). This reduction has direct clinical consequences. It is associated with deficits in memory, attention, and executive function, as well as increased suicide risk (58, 76, 94) This relationship may be mediated by structural brain differences, including reduced volume in ventromedial and insular regions involved in emotional regulation and interoceptive integration (95).

In this context, and from a modulatory perspective, emerging evidence suggests that behavioral factors such as physical exercise may partially influence this vulnerability. In particular, the BDNF Val66Met polymorphism has been associated with differential responses to exercise, with potential protective effects against post-traumatic symptoms, possibly mediated by epigenetic mechanisms such as changes in DNA methylation (96, 97). These findings support a broader conceptualization in which stress-related molecular alterations are integrated within an allostatic framework, where neuroendocrine and metabolic adaptations dynamically interact over time. (3, 98). Within this context, BDNF-related alterations may also contribute to broader neuropsychiatric manifestations, including the emergence of psychotic symptoms in PTSD, reflecting a more profound disruption of neural circuit stability (99). From a translational perspective, this molecular framework opens opportunities for the development of predictive and prognostic biomarkers. The integration of genetic susceptibility, epigenetic profiles, and clinical variables has shown potential in identifying individuals at higher risk of developing PTSD and in predicting symptom severity (100, 101). In parallel, the systemic consequences of chronic stress extend beyond the central nervous system. Inflammatory and metabolic dysregulation may contribute to peripheral conditions, including gastrointestinal disorders, through mechanisms involving microbiome alterations and persistent inflammation (102). Moreover, there is growing evidence that the biological impact of extreme stress may extend beyond the individual. Trauma-induced epigenetic modifications, particularly in genes such as FKBP5 and BDNF, may exert intergenerational effects, influencing behavioral and neurobiological outcomes in offspring of exposed individuals (91, 103). Similarly, emerging biomarkers such as circulating microRNAs may provide insight into resilience and long-term adaptation to stress, in addition to serving as potential therapeutic targets (104, 105).

Finally, non-pharmacological interventions, including sleep optimization and structured physical activity, have demonstrated the capacity to modulate this biological network, improving clinical outcomes and potentially reversing aspects of stress-induced dysregulation (106, 107). Importantly, these findings are consistent with the neurocognitive alterations summarized in **Table 2**, reinforcing the clinical relevance of BDNF dysregulation. However, variability in BDNF levels across studies suggests that its role is complex. In some cases, elevated peripheral levels may reflect compensatory responses rather than effective neuroprotection (60), highlighting the need to interpret BDNF alterations within a broader network context rather than as isolated markers.

#### 4.1.3 The endogenous anxiolytic modulator: neuropeptide Y

**Figure 6:**
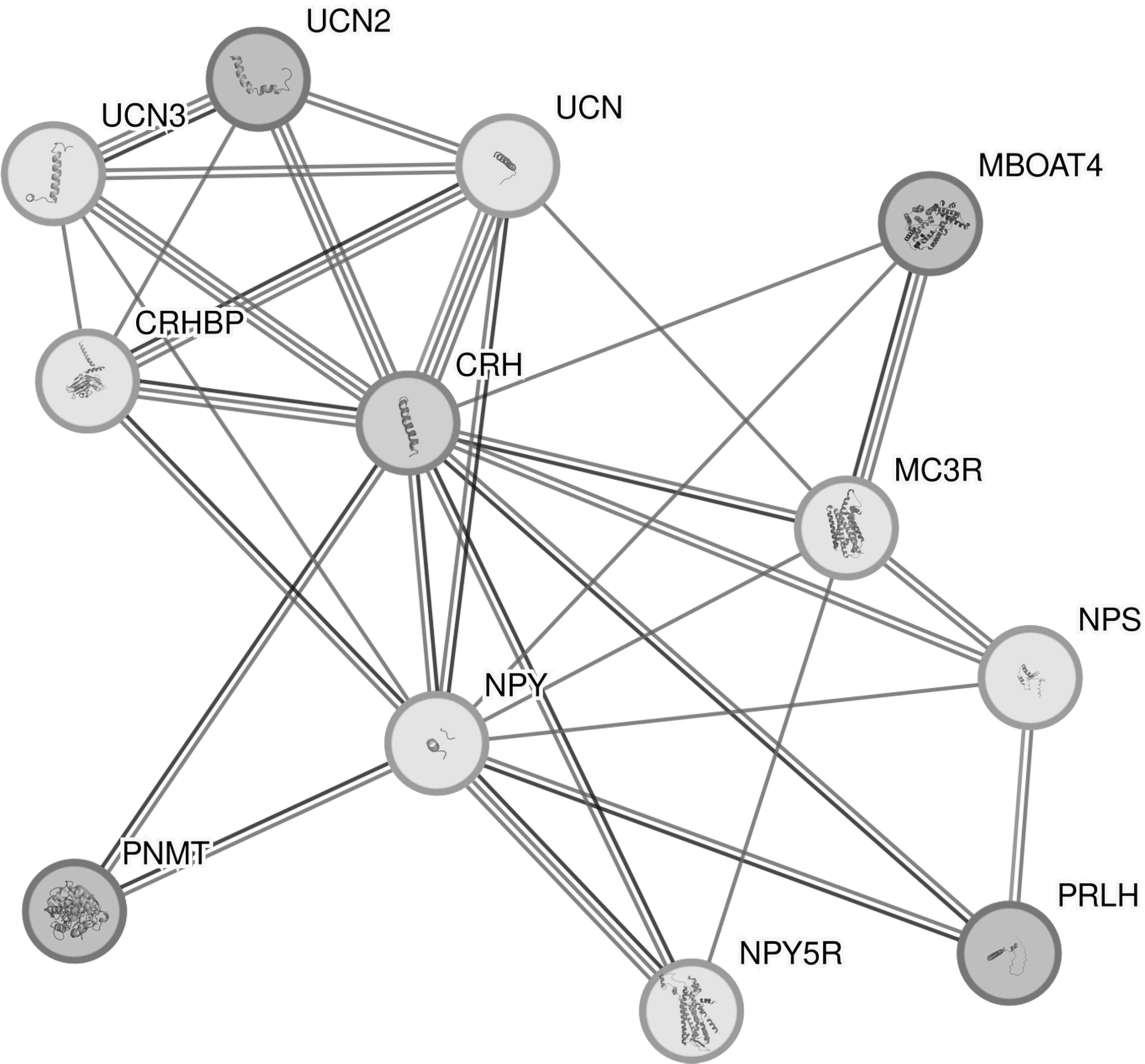
Neuroendocrine and peptidergic network associated with stress axis regulation and systemic homeostasis. The image represents key ligands, receptors, and enzymes that modulate the activation of the hypothalamic-pituitary-adrenal (HPA) axis and the circuits integrating stress, metabolism, and autonomic response. Corticotropin-releasing hormone (CRH) regulates the pituitary release of ACTH and can induce NLRP6 in the intestinal epithelium, modulating neuroimmunological interaction with the microbiota. The CRHBP binding protein binds to CRH and inactivates its signaling, preventing inappropriate adrenal stimulation. Urocortins (UCN, UCN2, and UCN3), members of the CRF family, act on CRFR1 and CRFR2 receptors to suppress food intake, delay gastric emptying, and contribute to post-stress homeostasis. Neuropeptide Y (NPY) and its NPY5R receptor regulate feeding behavior and energy balance through G protein-coupled receptors (GPCRs). The MC3R receptor mediates the effects of ACTH and MSH on circadian rhythmicity and metabolic adaptation. PNMT catalyzes the conversion of norepinephrine to epinephrine, integrating the sympathetic response. MBOAT4 activates ghrelin through acylation. PRLH stimulates prolactin, and NPS modulates arousal and anxiety. Collectively, this network coordinates allostasis in response to stress.

NPY plays a central role as an endogenous buffer against stress (see **Figure 6**). The results show that high NPY levels are associated with adaptive responses under acute stress conditions 61. In contrast, chronic PTSD is characterized by persistently reduced NPY levels in both plasma and CSF (63–65). This depletion has important consequences. It removes a key inhibitory mechanism on both the HPA axis and the sympathetic nervous system, thereby contributing to sustained stress reactivity. This mechanism is consistent with the increased cardiovascular and metabolic risk described in **Table 2** (69, 83). Thus, NPY functions as a bidirectional marker: it reflects resilience when dynamically regulated and vulnerability when chronically depleted.

#### 4.1.4 Inflammation as a consequence and driver of pathological stress: IL-6

**Figure 7:**
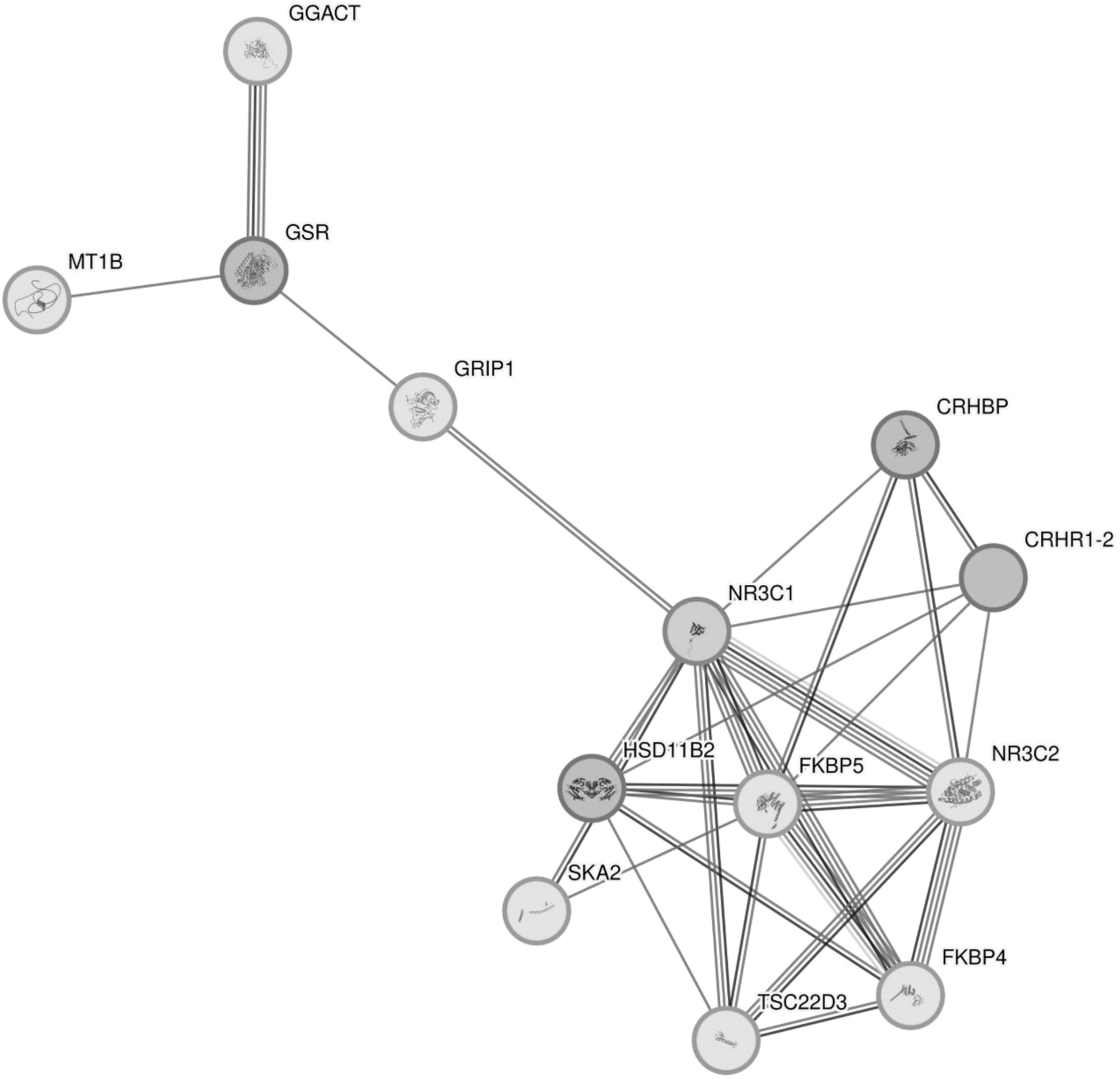
Functional network of immuno-inflammatory genes, redox regulators, and adaptive response modulators involved in tumor microenvironment remodeling. IGHV3-72 and IGHV3-43D encode immunoglobulin heavy chain variable regions essential for antigen recognition and B-lymphocyte clonal activation during humoral immunity. IL12A and IL12B constitute interleukin-12, a cytokine that stimulates T and NK cells and induces IFN-γ production. IL31 primarily activates STAT3 via the IL31RA/OSMR receptor, modulating inflammation and myeloid survival. IL-6 and TNF represent central nodes of systemic inflammation, regulating lymphocyte differentiation, acute-phase response, apoptosis, and cell proliferation. LBP enhances the recognition of bacterial lipopolysaccharide and amplifies cytokine release. CCL4L2 participates in leukocyte chemotaxis. TNFRSF6B acts as a decoy receptor that neutralizes FASL-mediated apoptotic signals. TBKBP1 intervenes in innate antiviral immunity through interaction with TBK1/IKBKE. GSR maintains reduced glutathione homeostasis and oxidative stress control. ARMS2 is associated with inflammatory susceptibility. Collectively, these genes articulate immunity, chronic inflammation, and redox regulation within contexts of glandular epithelial tumor progression.

The results further demonstrate that chronic stress is associated with sustained activation of inflammatory pathways, particularly through IL-6. Elevated IL-6 levels are consistently observed in PTSD and correlate with symptom severity (27, 28). This inflammatory state is not independent of neuroendocrine dysfunction. Instead, it reflects a disrupted interaction between the immune system and the HPA axis. Although cortisol normally suppresses inflammation, glucocorticoid resistance allows IL-6 levels to remain elevated. (see **Figure 7**) (74, 75). This persistent inflammation contributes to multiple systemic effects, including cognitive decline, metabolic dysfunction, and chronic pain, as summarized in **Table 2** (78, 81, 82).

#### 4.1.5 The integrated network: interconnections between the five molecules

**Figure 8:**
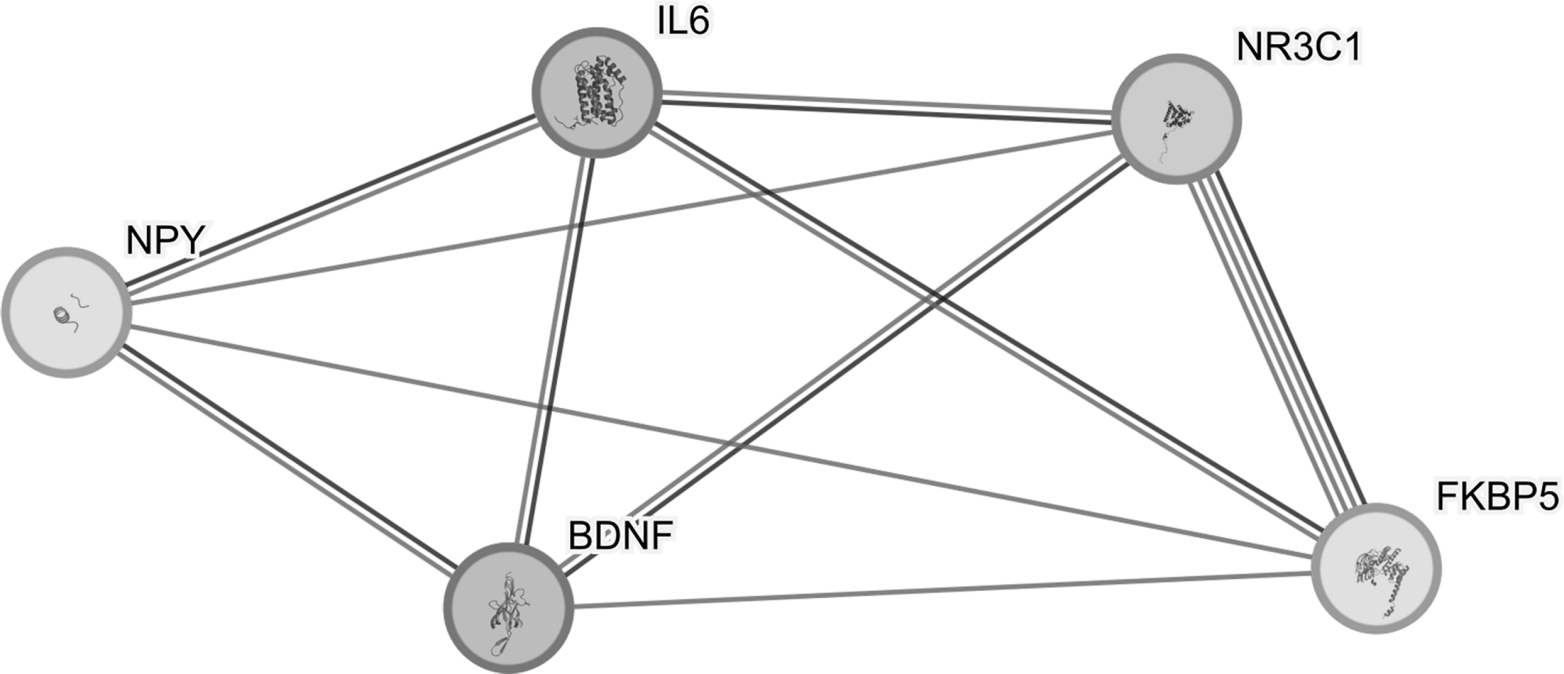
Integrative molecular network linking neuroendocrine signaling, systemic inflammation, and cellular plasticity in chronic stress contexts. IL-6 (Interleukin-6) is a pleiotropic cytokine and potent inducer of the acute-phase response; it participates in lymphocyte and monocyte differentiation, terminal maturation of immunoglobulin-producing B cells, and the generation of T(H)17 cells. It acts on immune cells, hepatocytes, hematopoietic progenitors, and central nervous system cells; as a myokine released following muscle contraction, it promotes lipolysis and improves insulin sensitivity.NR3C1 (Glucocorticoid Receptor, GR) functions as a ligand-dependent transcription factor that binds to glucocorticoid response elements (GREs) in nuclear and mitochondrial DNA, regulating inflammation, proliferation, and chromatin remodeling, and participating in selective mRNA degradation. FKBP5, an HSP90-associated co-chaperone, modulates GR sensitivity and regulates AKT signaling. BDNF, through NTRK2/TrkB, controls neuronal survival and plasticity. NPY integrates metabolic, autonomic, and neuroendocrine regulation. Together, these nodes articulate adaptive and potentially pathological responses to sustained stress.

A key contribution of this work is the integration of these findings into a unified network (see **Figure 8**).The results clearly indicate that these molecules interact in a coordinated manner rather than acting independently. FKBP5 and NR3C1 regulate HPA axis feedback. This, in turn, influences NPY release and sympathetic activation. NPY provides inhibitory feedback to both systems, but its depletion removes this control. At the same time, glucocorticoid resistance allows IL-6–mediated inflammation to persist. Finally, inflammation negatively affects BDNF, impairing neuronal plasticity. This interconnected structure explains how alterations in a single node can propagate through the system, generating the broad clinical phenotype observed in PTSD.

#### 4.1.6 Associated comorbidities and clinical implications

The results presented in **Tables 2** and **3** demonstrate that this network extends beyond PTSD and underlies multiple systemic comorbidities. Cognitive impairment is linked to the combined effects of reduced BDNF and increased IL-6 (76, 77). Suicidal behavior reflects the interaction between NPY depletion, BDNF alterations, and HPA axis dysregulation (59, 68, 79). Metabolic and cardiovascular disorders are driven by chronic inflammation and altered glucocorticoid signaling (3, 81). These findings support the concept of PTSD as a systemic disorder driven by a shared molecular network.

### 4.2 Most frequent cancers in military personnel and veterans: recent evidence from the military and veterans health systems

Military personnel and veterans represent a population with a distinct epidemiological profile shaped by occupational, environmental, and behavioral exposures. These include chemical agents, prolonged solar radiation, tobacco use, and sustained psychosocial stress. Rather than acting independently, these factors interact to produce a characteristic distribution of malignancies. The evidence synthesized in this study shows that prostate, lung, colorectal, melanoma, testicular, breast, cervical, and thyroid cancers constitute the core oncological burden in this population. Importantly, these tumors do not appear randomly distributed; instead, they follow identifiable patterns linked to demographic variables, healthcare access, and exposure history. Prostate cancer consistently emerges as the most prevalent malignancy among male veterans. Beyond incidence, recent studies highlight its clinical complexity, including the burden of metastatic castration resistant disease (5) and variability in outcomes across cohorts (85). The association between lifestyle factors and reduced risk (84) further suggests that this tumor reflects not only exposure history but also modifiable systemic influences. This has led to the proposal that veterans may represent a high-risk group requiring adapted screening strategies (86). Lung and bronchial cancer, in contrast, illustrate the direct impact of occupational and behavioral exposures. Their high incidence is strongly linked to tobacco use and environmental toxins, while the success of LDCT screening in reducing mortality demonstrates the value of targeted early detection (6). At the same time, survival differences observed in surgical studies (7) indicate that outcomes are also shaped by clinical and systemic factors beyond exposure alone. Colorectal cancer highlights the role of social determinants in shaping oncological outcomes. Variations in screening access, housing status, and healthcare continuity (8–10). reveal that disease burden is not solely biologically determined but also structurally mediated. This reinforces the need to integrate social context into cancer prevention strategies. Melanoma provides a clear example of exposure-driven risk, with cumulative ultraviolet radiation during military service acting as a key factor. However, methodological limitations in data capture (11) and gaps in preventive care (12, 13) suggest that its true burden may be underestimated, pointing to opportunities for improved surveillance and intervention. In younger populations, testicular cancer underscores the importance of survivorship. Although less frequent, its long-term metabolic and cardiovascular consequences (14) highlight how cancer burden extends beyond initial diagnosis. Among female veterans, breast and cervical cancers reveal additional layers of complexity related to access, equity, and prevention. Disparities in surgical outcomes (15) and variability in genetic testing access (16) indicate systemic gaps, while differences in screening adherence (18) emphasize the role of contextual factors in early detection. Thyroid cancer further expands this framework by introducing potential links between environmental exposure and molecular alterations. Evidence suggesting epigenetic changes associated with Agent Orange (20) raises the possibility that service-related exposures may leave lasting biological imprints relevant to tumorigenesis. Taken together, these findings indicate that the oncological burden in military populations is structured rather than random. It reflects the interaction between direct exposures and broader systemic processes, including aging, inflammation, and healthcare access. A key contribution of this study is the identification of a dissociation between tumor frequency and exposure intensity. While some cancers, such as lung cancer, align closely with occupational risks, others, such as prostate or colorectal cancer, show weaker exposure associations despite high prevalence. This discrepancy suggests that traditional models of carcinogenesis based solely on external exposures are insufficient to explain the observed patterns. Instead, the data support a stratified risk model in which environmental exposures interact with internal biological states shaped by chronic stress and inflammation. Within this model, neuroendocrine dysregulation particularly involving glucocorticoid signaling, FKBP5 activity, and IL-6-mediated inflammation emerges as a potential unifying mechanism. The convergence between epidemiological patterns and stress-sensitive molecular pathways provides a basis for integrating Phase 1 and Phase 2 findings. Specifically, the same neuroimmunoendocrine alterations implicated in PTSD may also modulate cancer susceptibility, progression, and clinical outcomes. This perspective supports the hypothesis of a stress inflammation oncogenesis axis, in which chronic activation of stress-response systems contributes to immune remodeling, metabolic dysregulation, and epithelial vulnerability. From this standpoint, cancer risk in military populations cannot be understood solely as the result of carcinogenic exposures but must also be viewed as the outcome of long-term biological adaptation to extreme stress. Within this framework, future research should focus on identifying the molecular intermediates that connect stress biology with tumor development. Integrating epidemiological data with biomarkers of neuroendocrine and inflammatory function may enable more precise risk stratification and targeted interventions. Ultimately, understanding cancer in military populations requires a shift from fragmented models toward an integrated approach that combines exposure history, systemic biology, and social determinants. This approach not only improves mechanistic understanding but also supports the development of more effective prevention, screening, and treatment strategies tailored to this unique population.

Despite the integrative nature of the proposed framework, several considerations warrant a more cautious interpretation of the available evidence. First, the relationship between chronic stress and cancer remains heterogeneous across studies, with some reports supporting a role for neuroendocrine and inflammatory dysregulation in tumor progression (27–32, 89, 90), while others demonstrate weaker or context-dependent associations after adjustment for confounding variables such as lifestyle factors, comorbidities, and healthcare access (8–10, 84–86). Second, most epidemiological data in military and veteran populations are observational, limiting causal inference and potentially affected by selection bias, exposure misclassification, and variability in cohort composition (5–10, 84–86). In addition, the observed associations may reflect the combined influence of social determinants and systemic health disparities rather than direct biological effects of stress alone (8–10, 18, 84–86). Third, tumor heterogeneity represents a critical limitation for generalization. The impact of chronic stress is unlikely to be uniform across all malignancies; rather, it may be more relevant in tumors that are highly responsive to endocrine and inflammatory signaling, such as hepatocellular carcinoma, pancreatic ductal adenocarcinoma, and prostate cancer (21, 25, 29–32, 51, 88–90), while other tumor types may be predominantly driven by direct carcinogenic exposures. Taken together (6, 11–13, 18–20), these considerations underscore the need to interpret the proposed stress–oncogenesis axis as a hypothesis-generating framework that integrates current evidence while acknowledging existing uncertainties and variability across biological and clinical contexts.

### 4.3 Extreme stress, the HPA axis, and glandular epithelial tumors related to NR3C1, FKBP5, IL-6, BDNF, and NPY

Glandular epithelial tumors, particularly hepatocellular carcinoma (HCC), pancreatic ductal adenocarcinoma (PDAC), and prostate adenocarcinoma, are highly sensitive to endocrine, inflammatory, and metabolic signaling, and in military populations exposed to extreme and prolonged stress, chronic activation of the hypothalamic–pituitary–adrenal (HPA) axis emerges as a central systemic driver that may favor malignant transformation. Within this framework, NR3C1 represents the principal effector of glucocorticoid signaling, and sustained receptor activation leads to broad transcriptional reprogramming affecting metabolic control, immune regulation, and cellular proliferation, while experimental evidence indicates that disruption of glucocorticoid signaling promotes hepatic steatosis and progression toward hepatocellular carcinoma, underscoring the requirement for tightly regulated NR3C1 activity (21). Importantly, chronic glucocorticoid exposure also induces stable epigenetic remodeling, including DNA methylation changes and chromatin reorganization, which may permanently alter transcriptional responsiveness in glandular tissues and thereby facilitate the transition from chronic inflammation to malignant transformation.

A second level of regulation involves co-chaperone systems such as BAG-1, which enhance anti-apoptotic signaling and stabilize glucocorticoid receptor activity (22). and under conditions of persistent stress this mechanism may contribute to clonal selection of epithelial cells with survival advantages, particularly within inflammatory microenvironments. In this context, although current interactomic databases do not show direct functional connectivity for genes such as ELOVL1, CLEC4M, and SLC17A5, this likely reflects incomplete literature coverage rather than true biological isolation, as supported by recent in silico analyses from our group including these genes and their interacting partners (LDHA and DNAJB5), which suggest their involvement in susceptibility and progression of glandular epithelial tumors (see **Supplementary Table 2**; manuscript in preparation), thereby defining a putative molecular module requiring experimental validation.

Within this broader network, FKBP5 emerges as a central integrator linking stress signaling to oncogenic pathways by modulating glucocorticoid receptor sensitivity and interacting with PI3K/AKT signaling, thereby influencing apoptosis, cellular survival, and therapeutic response (25, 26, 51). Its dysregulation is particularly relevant in hormone-sensitive tumors such as HCC and prostate cancer, where stress-induced overexpression may shift the balance toward proliferative signaling, whereas in pancreatic cancer characterized by constitutive AKT activation FKBP5 may further enhance intrinsic resistance mechanisms and tumor adaptability.

In parallel, inflammatory signaling through the IL-6/STAT3 axis constitutes a major mechanistic bridge between chronic stress and tumor progression, as IL-6 drives proliferation, metabolic reprogramming, and epitelial mesenchymal transition via STAT3 activation (29, 30). while also promoting loss of tissue homeostasis and acquisition of aggressive phenotypes in hepatocellular carcinoma (31). Moreover, IL-6 establishes autocrine loops that reinforce cancer stem cell properties and metastatic potential (32), and in conditions of chronic stress this signaling axis may be persistently amplified, particularly in tissues exposed to additional environmental or metabolic risk factors, a role further supported by its association with lung cancer proliferation (88).

Beyond inflammatory pathways, neurotrophic and neurovascular mediators further expand this model of tumor plasticity. BDNF signaling, acting through TrkB and the RAS/MAPK pathway, promotes cellular survival and phenotypic adaptation (33), and although classically studied in the nervous system, it also contributes to tumor cell resistance to apoptosis and stress adaptation, suggesting that its dysregulation under chronic stress may enhance tumor persistence and progression (34, 35).

Similarly, NPY functions as a neurovascular regulator within the tumor microenvironment, where Y1 and Y2 receptor signaling modulates vascular tone and promotes angiogenesis (37, 38), and sustained sympathetic activation may therefore facilitate tumor vascular remodeling and growth, with additional translational relevance given the potential of NPY receptor antagonists as anti-angiogenic strategies (39).

Integration of these pathways supports a unified model in which chronic stress orchestrates tumor development through coordinated neuroendocrine, inflammatory, and epigenetic mechanisms. Persistent activation of the HPA axis maintains glucocorticoid signaling via NR3C1, induces FKBP5 overexpression, and alters cellular stress sensitivity (23, 24), while simultaneous activation of the IL-6/STAT3 axis sustains inflammatory and proliferative signaling networks (89, 90). These converging processes promote epigenetic reprogramming, epithelial–mesenchymal transition, and expansion of cancer stem cell populations, which together define key hallmarks of aggressive and therapy-resistant tumors (40, 41), with additional modulation from BDNF- and NPY-mediated plasticity and vascular adaptation (37, 38).

**Figure 9:**
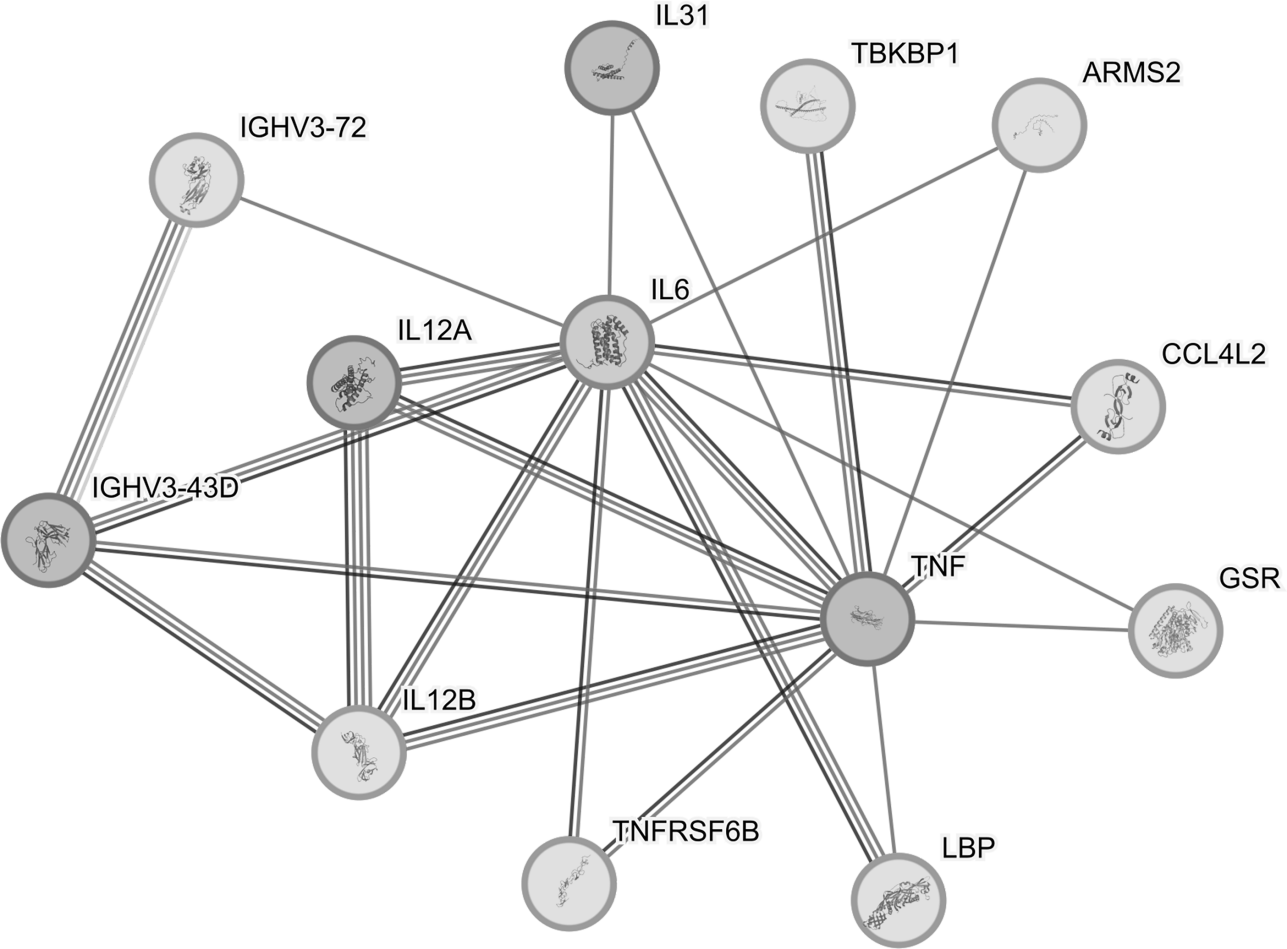
Schematic representation of the molecular convergence between genes associated with glandular epithelial tumors and extreme stress response genes. Nodes in blue correspond to genes with established functions in glandular epithelial tumorigenesis, including WNT pathway regulators (AMER1, AXIN1, CTNNB1, RNF43), chromatin modifiers (ARID1A), tumor suppressors (TP53, STK11), oncogenes (KRAS, BRAF, PIK3CA), telomerase (TERT), and energy metabolism factors (LDHA, ELOVL1, SLC17A5, CLEC4M, DNAJB5) (see **Supplementary Table 2**). Nodes in orange represent genes previously associated with the pathophysiology of extreme stress and neuroendocrine-immune regulation, specifically the glucocorticoid receptor (NR3C1), its co-chaperone (FKBP5), the neurotrophic factor BDNF, the neuropeptide NPY, and the pro-inflammatory cytokine IL-6. Connection lines represent functional or regulatory interactions documented in the literature. The integration of both gene sets into a single network supports the proposed model, in which chronic dysregulation induced by extreme stress may modulate key oncogenic pathways, favoring the progression of glandular epithelial neoplasms in military and veteran populations exposed to prolonged stress.

Taken together, these findings support the existence of a stress driven pathogenic axis in military populations that contributes to the initiation, progression, and aggressiveness of glandular epithelial tumors such as HCC, PDAC, and prostate cancer (**Figure 9**). More broadly, the data support a model in which sustained extreme stress functions as a cross-cutting biological modifier characterized by persistent neuroendocrine dysregulation, chronic inflammation, and impaired cellular adaptation. Importantly, the relevance of this framework lies not only in individual molecular pathways but in their dynamic and cumulative interaction, which may generate a vulnerable biological substrate capable of amplifying disease heterogeneity across exposed populations (27, 28, 44–50, 82).

This reshaping of the biological terrain is fundamentally driven by the mechanistic integration of the tumor microenvironment (TME) and systemic stress mediators. The persistent activation of the HPA axis and the resulting glucocorticoid resistance evidenced by the FKBP5/NR3C1 dysregulation facilitates a state of sterile inflammation (99, 101). In this context, the local TME is characterized by a high concentration of IL-6, which functions as a critical bridge between systemic stress and local tumor biology. This cytokine promotes an immunosuppressive landscape by activating the JAK/STAT3 pathway, which is known to recruit myeloid-derived suppressor cells (MDSCs) and polarize macrophages toward a pro-tumorigenic M2 phenotype, thereby weakening anti-tumor immunity (29, 30, 102). Furthermore, the interaction between systemic stress and local epithelial plasticity is reinforced by the roles of BDNF and NPY in the TME. We propose that under chronic stress, the loss of homeostatic NPY signaling and the overexpression of BDNF/TrkB not only drive the epithelial-mesenchymal transition (EMT) but also stimulate pathological angiogenesis (33–35, 37, 103). This mechanical depth explains how the systemic ‘attrition’ of the military environment creates a permissive niche for glandular epithelial neoplasms, where the synergistic effect of metabolic reprogramming (e.g., LDHA upregulation) and immune evasion fosters tumor progression and resistance to conventional therapies (104, 106).

From an epidemiological perspective, the non-random distribution of cancer types reinforces this interpretation, as the coexistence of malignancies with heterogeneous exposure dependencies suggests that classical carcinogenic models based solely on external risk factors are insufficient to explain observed patterns (5–10, 84–86). Accordingly, we propose that the interaction between stress dysregulation and chronic inflammatory signaling acts as a facilitating axis for cellular plasticity and tumor evolution, promoting aggressive phenotypes regardless of initiating exposure and integrating neuroendocrine and oncogenic processes within a continuous biological framework (23–26, 29–32, 89, 90).

In this context, this work advances the hypothesis that chronic extreme stress not only contributes to disease initiation but also actively reshapes the biological terrain in which tumorigenesis occurs, thereby redefining cancer risk as the emergent property of long-term systemic adaptation rather than isolated exposure events. This perspective opens new translational avenues aimed at validating stress-related molecular intermediates and targeting this systemic state as a complementary strategy for prevention and intervention in high-risk populations.

While this framework provides a novel integrative perspective, several limitations must be acknowledged. First, the proposed model is primarily hypothesis-driven, and while it is grounded in established neuroendocrine and inflammatory pathways (21, 23, 51), it requires further experimental and clinical validation to confirm the precise hierarchy of these interactions. Second, the heterogeneity of the evidence included ranging from molecular studies to large-scale military cohorts presents challenges in terms of data standardization (100, 104).

Furthermore, the epidemiological evidence discussed, although robust in identifying associations between chronic stress and cancer incidence in military populations (10–25, 84–86), is inherently limited in its capacity to support direct causal inference due to potential confounding variables. Finally, the component involving microgravity-derived gene convergence (e.g., LDHA and DNAJB5 modules) remains speculative (**Supplementary Table 2**). Although these genes show significant functional connectivity in silico, their role as definitive biomarkers for stress-induced glandular neoplasms under terrestrial conditions requires rigorous longitudinal assessment. Acknowledging these constraints is essential for a balanced interpretation of the integrative neuroendocrine-inflammatory framework presented here (See **Figure 9**).

## 5 Conclusion

Taken together, the evidence indicates that extreme and prolonged military stress does not merely constitute a psychopathological risk factor limited to the development of disorders such as PTSD; rather, it represents a systemic biological determinant capable of inducing lasting reprogramming of the neuroendocrine, inflammatory, and epigenetic axes. This reprogramming is articulated through a hierarchical dysregulation of the hypothalamic–pituitary–adrenal (HPA) axis, characterized by persistent activation of the glucocorticoid receptor (NR3C1), modulatory overexpression of FKBP5, chronic inflammatory activation mediated by IL-6/STAT3, and alterations in cellular plasticity and neurovascular signaling dependent on BDNF and NPY.

Far from being confined to the neuropsychiatric domain, this dysregulated neuroimmunoendocrine network configures a state of pathological allostasis that transcends the central nervous system, affecting peripheral tissues sensitive to hormonal and inflammatory signaling. In this context, a systemic microenvironment is established characterized by low-grade inflammation, sustained epigenetic remodeling, metabolic alterations, and selective advantages for resilient cellular clones. These conditions may facilitate the progression of glandular epithelial neoplasms prevalent in military and veteran populations, such as hepatocellular carcinoma, pancreatic adenocarcinoma, and prostate cancer.

Consequently, extreme stress must be understood as a systemic biological modulator with psychiatric, metabolic, and potentially oncogenic implications. This integrative framework redefines PTSD not merely as an isolated clinical entity, but as the visible manifestation of a broad molecular disruption that may contribute to the oncological burden observed in veterans. Therefore, the stress-induced neuroimmunoendocrine axis emerges as a central yet underestimated, pathogenic component at the intersection of military medicine, biological psychiatry, and translational oncology.

## Data Availability

The original contributions presented in the study are included in the article/**Supplementary Material**. Further inquiries can be directed to the corresponding author.
